# Methadone vs. buprenorphine/naloxone during early opioid substitution treatment: a naturalistic comparison of cognitive performance relative to healthy controls

**DOI:** 10.1186/1472-6904-7-5

**Published:** 2007-06-12

**Authors:** Pekka Rapeli, Carola Fabritius, Hannu Alho, Mikko Salaspuro, Kristian Wahlbeck, Hely Kalska

**Affiliations:** 1 Unit for Drug Dependence, Department of Psychiatry, Helsinki University Central Hospital, Box 590, FIN-00029 Helsinki, Finland; 2 Unit on Prevention and Treatment of Addictions, Department of Mental Health and Alcohol Research, National Public Health Institute (KTL), Finland; 3 Research Unit of Substance Abuse Medicine, University of Helsinki, Finland; 4 Department of Psychology, Faculty of Behavioural Sciences, Helsinki, Finland; 5 National Research and Development Centre for Welfare and Health STAKES, Finland and Psychiatric Unit, Vaasa Central Hospital, Vaasa, Finland

## Abstract

**Background:**

Both methadone- and buprenorphine-treated opioid-dependent patients frequently show cognitive deficits in attention, working memory, and verbal memory. However, no study has compared these patient groups with each other during early opioid substitution treatment (OST). Therefore, we investigated attention, working memory, and verbal memory of opioid-dependent patients within six weeks after the introduction of OST in a naturalistic setting and compared to those of healthy controls.

**Methods:**

The sample included 16 methadone-, 17 buprenorphine/naloxone-treated patients, and 17 healthy controls matched for sex and age. In both groups buprenorphine was the main opioid of abuse during the recent month. Benzodiazepine codependence, recent use, and comedication were also common in both patient groups. Analysis of variance was used to study the overall group effect in each cognitive test. Pair-wise group comparisons were made, when appropriate

**Results:**

Methadone-treated patients, as a group, had significantly slower simple reaction time (RT) compared to buprenorphine/naloxone-treated patients. In Go/NoGo RT methadone patients were significantly slower than controls. Both patient groups were significantly debilitated compared to controls in working memory and verbal list learning. Only methadone patients were inferior to controls in story recall. In simple RT and delayed story recall buprenorphine/naloxone patients with current benzodiazepine medication (*n *= 13) were superior to methadone patients with current benzodiazepine medication (*n *= 13). When methadone patients were divided into two groups according to their mean dose, the patient group with a low dose (mean 40 mg, *n *= 8) showed significantly faster simple RT than the high dose group (mean 67 mg, *n *= 8).

**Conclusion:**

Deficits in attention may only be present in methadone-treated early phase OST patients and may be dose-dependent. Working memory deficit is common in both patient groups. Verbal memory deficit may be more pronounced in methadone-treated patients than in buprenorphine/naloxone-treated patients. In sum, to preserve cognitive function in early OST, the use of buprenorphine/naloxone may be more preferable to methadone use of, at least if buprenorphine has been recently abused and when benzodiazepine comedication is used. Longitudinal studies are needed to investigate if the better performance of buprenorphine/naloxone-treated patients is a relatively permanent effect or reflects "only" transient opioid switching effect.

## Background

In opioid substitution treatment, the opioid-dependent patient receives long-acting mu opioid receptor agonists in order to prevent withdrawal symptoms and to reduce craving for street opioids. The full mu opioid agonist methadone is the most commonly used drug in OST programs. If overdosed, which may happen in cases of abuse, methadone may cause fatal respiratory depression. Therefore, partial mu opioid receptor agonist and kappa receptor antagonist buprenorphine, with a ceiling effect on respiratory depression, has been increasingly used in OST programs. Buprenorphine, however, is commonly abused in several countries; and in combination with other psychoactive substances it may also be hazardous [1,2]. Therefore, a safer drug combining buprenorphine and naloxone has been developed. The compound contains buprenorphine and naloxone in 4:1 ratio, and if used sublingually, it has practically equal pharmacokinetic properties as buprenorphine alone [3,4].

Already during the first few weeks of their OST patients often show reduction of use of illegal opioids and related problem behaviors [5,6]. Some patients, however, experience negative treatment effects including cognitive disturbances and relate them to their OST drug. This needs to taken seriously as drug-dependent patients experiencing troubles in concentrating and remembering have poor treatment engagement and treatment prognosis [7,8]. Thus, it is relevant to study objective cognitive function of early OST patients.

When given to healthy volunteers, both methadone and buprenorphine have shown adverse effects on attention and memory [9,10]. When these drugs are given to opioid-dependent patients, their cognitive effects may be different because these patients have tolerance for opioids. As an example of this, a single dose of methadone (5 or 10 mg) slowed down simple RT of healthy volunteers but had no such an effect on methadone-treated opioid-dependent patients in stabilized treatment (a stable methadone dose regimen from 20 mg to 70 mg for at least one month) [11]. In the same vein, a one third increase in methadone did not affect memory or RT performance of opioid-dependent patients who had been in treatment at least for 6 months [12]. However, switching from opioid of abuse to different opioid for OST purposes may cause transient cognitive side-effects. In accordance with this idea heroin abusing opioid-dependent patients studied during the first week of methadone-aided withdrawal treatment showed verbal memory deterioration after the full methadone dose of 35 mg on average compared to placebo or to halved dose [13].

Cognitive effects of buprenorphine in OST patients are not well known. Some evidence exists for buprenorphine having less adverse effect on driving-related attention than methadone [3,4,14,15]. However, in a recent comparison, made after 12 months of OST, both buprenorphine and methadone-treated patients were inferior to controls in visual memory [16].

In clinical settings, OST patients typically have used, and may still use, other substances of abuse. Benzodiazepine abuse is particularly common among individuals starting OST [17]. At the same time other psychoactive drugs are often prescribed to them. Yet, few studies have dealt with this issue. For instance, the studies of Soyka et al., which showed better performance among buprenorphine-than among methadone-treated OST patients, mainly describe patients with current polysubstance abuse and psychoactive polytherapy [14,15].

In sum, both methadone and buprenorphine/naloxone as such or in combination with other psychoactive medications may have negative effect on cognition in OST patients. To our knowledge, no study has addressed this issue in a naturalistic clinical sample of patients who are starting their OST – a period when cognitive deficits might be pronounced. Therefore, we evaluated attention, working memory, and verbal memory of methadone- or buprenorphine/naloxone-treated patients starting OST and compared these to those of controls.

## Methods

### Participants

The study participants with opioid dependence were volunteers from a consecutive series of patients accepted for standard OST in the addiction clinics of Helsinki area. The introduction of combined buprenorphine/naloxone OST in Helsinki in 2004 enabled a comparison of cognitive abilities between the methadone-treated and the buprenorphine/naloxone-treated patients. Healthy control participants were recruited from adult education centers and by word of mouth.

The inclusion criteria for all participants were age between 18 – 50 years. The additional inclusion criteria for OST patients were opioid dependence according to DSM-IV, and the start of OST during the last six weeks. We excluded participants with current uncontrolled polysubstance abuse, acute alcohol abuse, or acute axis I psychiatric morbidity according to Diagnostic and Statistical Manual of Mental Disorders (DSM-IV) other than substance abuse disorders. Also we excluded participants with severe brain injury, chronic neurological disease, history of other than substance-induced psychoses, epileptic seizures, human immunodeficiency virus (HIV) infection, pregnancy, or primary cognitive deficit. Each opioid-dependent participant eligible for our study was screened by urine sample for substance abuse on the day of testing and at least once in the preceding week. Healthy controls were selected for substance abuse screening at random and we screened one third of them. After excluding participants showing positive drug screen on the day of testing we included 16 methadone-, 17 buprenorphine/naloxone-treated patients, and 17 healthy controls.

The study protocol was accepted by the Ethics Committee of Helsinki University Central Hospital. We obtained a written informed consent according to the Declaration of Helsinki from all participants, and paid them € 60 if they attended all the necessary visits.

Table 1 shows major demographic variables of each group. When appropriate, we performed pair-wise group comparisons with analysis of variance (ANOVA) or with chi-square-test. The groups did not differ in age or sex distribution. The OST groups did not differ in history of substance abuse or duration of OST. As shown in Table 1 the control group had more education than the patient groups. The control group had superior verbal intelligence (Verbal IQ) relative to the methadone patients but not to the buprenorphine/naloxone patients. The main opioid of abuse within the last month was buprenorphine among all participants of the buprenorphine/naloxone group and among most participants (75%) in the methadone group. There were four cases (25%) of recent month abuse of heroin or methadone in the methadone-treated group. Also participants in the buprenorphine/naloxone group reported, however, earlier periods of heroin or methadone abuse. As expected, nearly all opioid-dependent participants had used also other substances of abuse within the last month. In general, these non-selected opioid-dependent participants represent reasonably well current opioid abusing populations in Finland where buprenorphine has become the main street opioid [18]. There has been a similar trend for increase of buprenorphine abuse world-wide [2].

**Table 1:** Group demographics

	Methadone (*n *= 16)	Buprenorphine/Naloxone (*n *= 17)	Control (*n *= 17)	Group comparison *p*-values
Age, years (*M, SD*)	30.8 (8.8)	28.1 (6.3)	31.1 (11.2)	*ns*
Sex: females/males	9/7	7/10	9/8	*ns*
Verbal intelligence ^a ^(*M, SD*)	98.4 (8.7)	102.4 (8.4)	105.4 (9.8)	C > M*
Education, years (M, SD)	10.4 (2.0)	11.1 (2.2)	13,0 (1.7)	C > M** C > BN**
Dependencies				
Opioid	100%	100%	-	*ns*^*b*^
Alcohol	0%	6%		*ns*^*b*^
Amphetamine	0%	11%		*ns*^*b*^
Benzodiazepines	100%	89%		*ns*^*b*^
Cannabis	6%	11%		*ns*^*b*^
Main opioid of abuse used within last month (%)				
Buprenorphine	75%	100%	-	*ns*^*b*^
Heroin	13%	0%		*ns*^*b*^
Methadone	13%	0%		*ns*^*b*^
Other substances of abuse used within last month (%)				
Alcohol (heavy use)^c^	6%	17%	6%	*ns*
Amphetamine	19%	29%	0%	*ns*
Benzodiazepine^d^	94%	94%	0%	M & BN > C**
Cannabis	38%	24%	0%	M > C*
Nicotine (daily use)	100%	100%	35%	M & BN > C**
Duration of opioid substitution treatment in the day of testing, days (M, SD)	14.3 (7.4)	11.0 (8.1)	-	*ns*^*b*^
Duration of opioid abuse, years (M, SD)	12.1 (7.7)	10.0 (3.5)	-	*ns*^*b*^
Duration of any substance abuse, years (M, SD)	16.9 (8.7)	15.7 (5.0)	-	*ns*^*b*^

^a^Estimation based on WAIS-R Vocabulary score.

^b^Tested only between methadone- and buprenorphine/naloxone-treated patients.

^c^Alcohol use was considered heavy if it was at least mean weekly 16 portions (12 g) for females and 24 weekly portions for males.

^d^Includes benzodiazepines used on prescription.

M = methadone, BN = buprenorphine/naloxone

> = superior than, *** = statistically significant at level *p *< 0.001. ** = statistically significant at level *p *< 0.01. * = statistically significant at level *p *< 0.05.

### Procedure

Cognitive testing was done three to six hours after the administration of opioid substitution drug, i.e. when drug plasma concentration is known to be highest [19]. Under supervision, the methadone patients were given a mean dose of 53.4 mg (*SD *= 18.6) of methadone, range 30 – 105 mg, in liquid form on the test day morning. The buprenorphine/naloxone patients, also under supervision, received a mean dose of 15.8 mg (SD = 3.2) of buprenorphine and 3.9 mg (SD = 0.8) of naloxone (range 8 – 24 mg of buprenorphine and 2 – 6 mg of naloxone) as a sublingual tablet. In this naturalistic study, all participants received their prescribed psychoactive medications on the test morning according to their clinical dose regimen. After that, however, opioid withdrawal syndrome relievers were given according to individual needs of the patients. Medications taken by the patients in the 24 hour period before the test are described in Table 2. Notably, all participants in both groups were either dependent on benzodiazepines, had abused them during last month, or were given them as a part of their early OST medications. In order to estimate the current benzodiazepine doses of the groups, all benzodiazepines were converted to diazepam equivalent doses according to the Ashton table [20]. In the cases of nitrazepam and temazepam the diazepam equivalent doses were halved in order to account for their use as hypnotics prior the night before testing. After this conversion no statistically significant difference existed between the patient groups in their mean estimated diazepam equivalent dose, 23.0 mg (SD = 20.2) in the methadone group and 19.6 mg (SD = 14.2) in the buprenorphine/naloxone group.

**Table 2:** Comedications among OST patients within the last 24 h before testing

Medications used within 24 hours of test	Methadone-treated patients (*n *= 16)		Buprenorphine/Naloxone- treated patients (*n *= 17)	
	Proportion of patients	Dose, range	Proportion of patients	Dose, range

Antidepressives (any)	44 %		35'%	
Essitalopram	6%	5 mg		
Citalopram	6 %	20 mg		
Doxepine			12%	75 – 100 mg
Fluoxetine	13%	20 – 30 mg		
Mirtazapine	13%	15 mg		
Paroxetine			6%	50 mg
Sertraline	6%	50 mg	12%	50 mg
Venlaflaxine			12%	75 mg
Anxiolytics, sedatives and hypnotics:				
Benzodiazepines (any)	81 %		76%	
Diazepam	38%	5–20 mg	29%	15 -40 mg
Oxazepam	44%	45 – 120 mg	47%	30 – 90 mg
Nitratzepam*	6%	20 mg		
Tematzepam *	19%	20 mg	12%	20 mg
Non-benzodiazepine hypnotics (any)	25%		35%	
Zolpidem *	6%	10 mg	6%	15 mg
Zopiclone *	19%	7.5 – 15 mg	24%	7.5 – 15 mg
Neuroleptics † (any)	25%		18%	
Chlorpromazine			6%	50 mg
Flupenthixole	6%	0.5 mg		
Levomepromazine	6%	150 mg	6%	100 mg
Quetiapine	13%	50–100 mg	6%	300 mg
Opioid withdrawal symptom or pain relievers (any)	69 %		53%	
Hydroxyzine	38%	25–200 mg	24%	75 – 300 mg
Ibuprofeine	13%	600– 2400 mg	6%	400 mg
Lofexidine	6%	0.2mg	18%	0.2 – 0.6 mg
Metoclopramide	6%	10 mg		
Naproxen	6%	500 mg	6%	500 mg
Propranol	6%	20 mg		
Valproate	13%	500 – 1000 mg	24%	500 – 1000 mg
None medication	13%		12%	

* Used as a hypnotic the night before testing.

† Used with anxiolytic indications

### Cognitive tests

A battery of cognitive tests included tests of attention, working memory, and verbal memory.

**Attention **was assessed by the Alertness and Go/NoGo- tasks from the Test for Attentional Performance (TAP) which include computer software and RT key-pad. [21]. In the Alertness task, visual RT was assessed with and without preceding auditory warning signal. The without signal condition of the Alertness test is a simple RT task, and is thought to reflect tonic or intrinsic alertness [22]. The with signal condition is thought to reflect both tonic and phasic alertness. The conditions were presented in the A-B-B-A – order. The Go/NoGo condition assessed integrity of response-selection and executive control of attention [23,24]. Visual stimuli were presented one by one. For two stimuli out of five an instant reaction is required, and for the others a reaction needs to be inhibited. Reaction times and correctness of responses were recorded. In all the TAP tests median of RTs was used as a RT parameter.

**Working memory **was assessed by the Letter-Number-Sequencing task (LNS) from the Wechsler Memory Scale-third version (WMS-III) and by the computerized version of the Paced Auditory Serial Addition Task (PASAT) from the FORAMENRehab software package [25-27]. The LNS assesses verbal working memory storage added with processing demand. In the PASAT complex working memory functions required are continuous storage of previous number, rapid arithmetical processing, and executive control of interference from previous items or from ongoing adding process. In our study presentation rate of a new number to be added to the previous one was set as one in every 1.6 second.

**Verbal memory **was assessed by a list learning task and by a story recall task: the Memory for Persons Data (MPD) and The Logical Memory (LM), respectively [25,28,29]. Both tests were presented in modified versions. In the LM, which is a subtest of the WMS-III, only one story was presented and recalled immediately and again after 30 minutes. In the MPD only three persons, each with 5 items, were presented. First there were two learning trials with immediate recall. If the participant could recall all 15 items correctly in both trials no more learning trials were administered. If this condition was not met, there were additional trials until the participant was able to recall all the items correctly in two consecutive trials. A maximum of four trials were administered. After five minutes recall of the items was asked for and possible errors were corrected for. Finally, after 30 minutes delayed recall of all the items was asked for.

### Statistical analysis

Analysis of variance (ANOVA) was used to study the overall group effect in each cognitive test. This was followed, when appropriate, by pair-wise group comparisons. We used multiple planned ANOVAs because comparisons were aimed at each variable separately. In all analyses, statistical significance was set at 0.05 (two-tailed). For each variable we corrected multiple pair-wise comparisons by Holm's procedure. We examined homogeneity of variances in each measure by Levene's test. In the simple RT and the MPD first trial performance, the data was first transformed by reciprocal or logarithmic transformation to normalize the distributions. For the both of the Go/NoGo conditions, the MPD last two last learning trials, and in the MPD delayed recall the distributions could not be normalized. First, we analyzed these results by non-parametric Kruskal-Wallis ANOVA, which then were followed, when appropriate, by pair-wise Mann-Whitney U test. We did not covary for the group difference in education favoring the control group over methadone group. This was based on the contention that the assumption of similar linear relation between education and cognitive performance in both groups needed for analysis of covariance (ANCOVA) was not met. All participants with opioid dependence had started substance abuse in their early teen years. Once the substance abuse history begins it soon affects educational achievement by class non-attendance etc. So, years of education does not reflect cognitive ability in this populations similarly to the general population [30]. However, in the second phase of the analysis, in order to evaluate the role of premorbid intellectual factors, we set verbal IQ as a covariate for other measures than RT measures. The association between simple RT measures and intelligence is weak and may not be linear [31]. Demographic data was studied as pair-wise group comparisons without first requiring significant overall group effect. Statistical analyses were done by SPSS statistical software, version 13.0, with an exception of the effect size calculations. For this purpose an effect size calculator provided by Durham University, UK was employed [32]. In these analyses we used pooled samples and corrected the values by Hedge's correction for small sample bias.

## Results

In the attention domain there were significant overall group effects in two tasks: in the simple and Go/NoGo RTs (*F*_2,47 _= 4.77, *p *= 0.013; χ^2^_2 _= 6.39, *p *= 0.041, respectively). In working memory, significant group effects were found in both tasks employed; the LNS and the PASAT (*F*_2,46 _= 11.99, *p *= 0.0001, *F*_2,46 _= 7.81, *p *= 0.001). In verbal memory the group effect was significant in the verbal list learning as measured by the MPD first trial and the MPD delayed recall, and also in the immediate story recall as measured by the LM (*F *_2,47 _= 7.29, *p *= 0.002, χ^2^_2 _= 9.24, *p *= 0.01, *F *_2,47 _= 5.49, *p *= 0.007). Table 3 shows group performances in each cognitive test, along with statistical analyses of pair-wise ANOVAs or Mann-Whitney U tests. As seen in Table 3 buprenorphine/naloxone patients were superior to methadone patients in the simple RT. The buprenorphine/naloxone patients showed no difference in attention tests compared to the control group. Rather surprisingly, buprenorphine/naloxone patients were slightly, but not statistically significantly, faster than controls in simple RT. In this task their performance variance was also reduced compared to other groups, which was confirmed by Levene's test of equality of variances (*F*_2,47 _= 4.13, *p *= 0.022). Both methadone patients and controls showed improvement of performance when RT was performed after warning signal whereas buprenorphine/naloxone patients showed no such improvement. In the Go/NoGo RT, controls were superior to methadone patients. In working memory tests controls were superior to both groups of OST patients. In verbal memory controls were superior to both patient groups in the MPD first learning trial. In the immediate LM controls were superior to methadone patients.

**Table 3:** Groups comparisons of cognitive measures using ANOVA

**Domain Test**	**Methadone (*n *= 16)**	**Buprenorphine/Naloxone(*n *= 17)**	**Control(*n *= 17)**	**Statistical comparisons between groups showing better performance first**	**Effect size (Cohen's d)**
	**Mean ± SD**	**Mean ± SD**	**Mean ± SD**		

Attention					
TAP Tonic Alertness, simple reaction time	257.6 ± 32.1	228.0 ± 13.0	244.4 ± 30.0	BN < M **	1.11
TAP Phasic Alertness, reaction time after warning signal	245.6 ± 30.4	227.4 ± 17.0	230.3 ± 31.7		
TAP Go/NoGo, reaction time	528.3 ± 82.0	496.9 ± 65.3^a^	465.5 ± 39.5	C < M*	0.88
TAP Go/NoGo, errors	0.6 ± 0.7	1.2 ± 1.4^a^	0.5 ± 0.6		
Working memory					
WMS-III LNS	8.8 ± 2.6^b^	8.7 ± 1.7	11.8 ± 3.1	C > M** C > BN**	1.02 1.21
PASAT	34.9 ± 10.6^b^	31.3 ± 10.8	47.8 ± 9.3	C > M** C > BN***	1.27 1.60
Memory					
MPD, first trial	10.1 ± 3.0	10.6 ± 2.4	13.0 ± 1.4	C > M** C > BN*	1.22 1.19
MPD, sum of two last trials	14.6 ± 1.0	14.8 ± 0.4	14.9 ± 0.2		
MPD, delayed recall	13.9 ± 1.0	14.2 ± 1.0	14.8 ± 0.4	C > M** C > BN*	
WMS-III logical memory, immediate recall	12.5 ± 2.9	14.3 ± 3.6	16.3 ± 3.4	C > M**	1.17
WMS-III logical memory, delayed recall	11.1 ± 4.3	13.4 ± 3.3	14.5 ± 4.1		

TAP = Test for Attentional Performance;

PASAT = Paced Auditory Serial Addition Task;

WMS-III = Wechsler Memory Scale-third version;

MPD = Memory for Persons Data.

C = control, M = methadone, BN = buprenorphine/naloxone

*** = statistically significant at level *p *< 0.001.

** = statistically significant at level *p *< 0.01. * = statistically significant at level *p *< 0.05.

^a^*n *= 16.

^b^*n *= 15.

In order to investigate if group differences were due to differences in premorbid intelligence between the groups, the Verbal IQ estimate was set as a covariate for all tasks with verbal stimuli. All statistically significant group differences remained significant after adjusting for the covariate. Statistically significant group by Verbal IQ interactions were not found.

**Figure 1:**
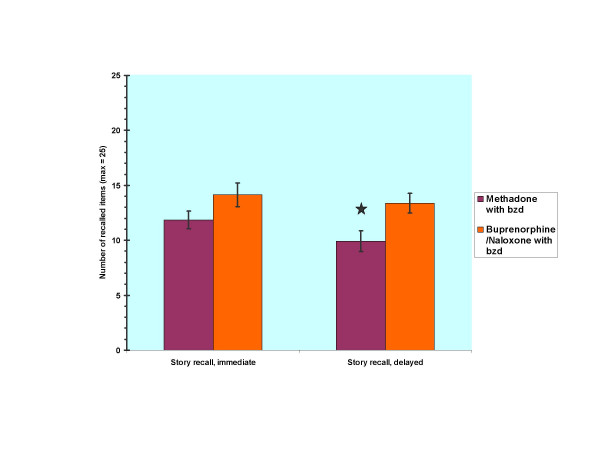
Story recall performance of methadone- vs. buprenorphine/naloxone-treated patients with benzodiazepine (bzd) comedication. * = *p *< 0.05

Post hoc analyses were done in order to investigate the role of benzodiazepine comedication on cognitive performance between the OST drug groups. In these analyses patients without current benzodiazepine medication were excluded leaving 13 patients in both groups. After exclusion all participants in both groups were dependent on benzodiazepines and had used them within the last month before the OST. The mean dose in the methadone group was 54.2 mg (*SD *= 18.7) of methadone and 28.3 mg (*SD *= 18.6) of diazepam equivalent. The mean dose in the buprenorphine/naloxone group was group was 16.3 mg (*SD *= 2.9) of buprenorphine and 25.6 mg (*SD *= 10.1) of diazepam equivalent. For demographic variables there no significant differences emerged between groups. The group with buprenorphine/naloxone and benzodiazepine medication was superior to the methadone group with benzodiazepine medication in simple RT (*U *= 38.00, *p *= 0.017) and in delayed recall of the LM (*F*_1,24 _= 6.15, *p *= 0.021, d = 0.94)). Figure 1 depicts group performances in both conditions of the LM.

**Figure 2:**
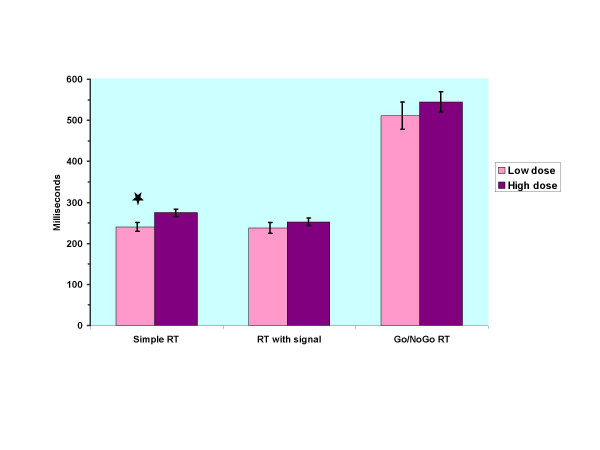
Comparison of high vs. low methadone dose groups in reaction times (RT). * = *p *< 0.05

Finally, post hoc analyses were made to study the role of OST doses on cognitive performance. For these analyses we split the patients groups into low and high dose groups depending on their median OST drug dosage. After this division the mean doses of methadone in the low dose (*n *= 8) and high dose group (*n *= 8) were 40.0 mg (*SD *= 5.3) and 66.9 mg (*SD *= 17.3). Among buprenorphine/naloxone patients nine patients received the same dose of 16 mg, and very few cases fell in the tails of the dose distribution. Therefore, dose analyses were restricted to methadone patients. Patients with low a methadone dose had faster RTs in all conditions than patients with high dose. Figure 2 depicts these differences. In the simple RT the difference reached statistical significance (*p *= 0.025, d = 1.19). The mean simple RT time in the low methadone dose group was 240.3 ms (SD = 29.9), which was very close to the performance of the control group. No significant differences between the groups were found in demographic variables except in days in the OST. The low dose group had been fewer days on OST medication, mean 8.6 days, and high dose group, mean 20.0 days (*SD *= 1.9 vs. 6.3 respectively, p < 0.001). No significant differences between the groups emerged in other psychoactive medications. In the low dose group 75% of the participants had benzodiazepine and 25% had neuroleptic medication, the corresponding figures being 88% and 25% in the high dose group. In the low dose group 38% received hydroxyzine vs. 25% in the high dose group. Conversion of long or intermediate acting benzodiazepines to a diazepam equivalent dose neither showed significant difference between the groups, mean dose for the low dose group being 29.6 mg (*SD *= 28.7) and 18.1 in the high group (*SD *= 10.9).

## Discussion

In this first study comparing cognitive function of methadone- and buprenorphine/naloxone-treated patients during early OST both patient groups were inferior to controls in working memory and verbal memory. Methadone-treated patients showed inferior performance also in attention and more deficits in verbal memory. In the attention task measuring alertness methadone-treated patient were inferior to buprenorphine/naloxone-treated patients. The effect sizes of group differences in comparison to controls in working memory and verbal memory were close to the ones obtained in other studies of opioid-dependent populations [33-35]. In attention tasks studies with stabile OST patients the effect sizes have been variable depending on specific tasks used [36].

Working memory performance, which was inferior in both patient groups, was measured by the PASAT and the LNS tasks combining maintenance with organization of material or with interference. Efficient performance of these tasks depends on activation of widespread neural networks including bilateral frontal and parietal cortices. Especially pronounced activity is seen in right hemisphere [37,38]. Methadone-treatment of opioid-dependence reduces cerebral blood flow (CBF) particularly in frontal cortices and the patients often have left-greater-than-right CBF asymmetry [39]. There are no similar studies concerning buprenorphine treatment though buprenorphine administration has been shown to reduce brain CBF in substance abusing population [40]. In general, the CBF reductions associated with opioid-dependence are probably linked to inadequate energy supply to the brain and changes in releases of several neurotransmitters such as catecholamines and acetylcholine [41,42]. Catecholamines are important for integrity of working memory, while acetylcholine is important also for learning and memory consolidation. [43,44]. Thus, it does not astonish that both OST groups showed inferior verbal list learning performance relative to controls. Methadone patients' performance was reduced also in immediate story recall.

After excluding patients without benzodiazepine medication the difference between the methadone and buprenorphine group in delayed story recall appeared statistically significant and showed a large effect size. Thus, it is possible that full mu opioid agonist methadone disrupts more acetylcholine release and consequently impairs more verbal memory than partial mu opioid agonist buprenorphine. Other factors that may be involved in memory deficits of the OST patients are alterations of glutamatergic synapses after chronic opioid administration or inhibition of new cell formation in the hippocampus after chronic mu opioid receptor activation [45,46].

Methadone patients were slower than buprenorphine/naloxone patients in simple RT reflecting alertness and slower than controls in the Go/NoGo RT task reflecting response selection and executive control of attention. On test day methadone patients received a mean dose of 23 mg diazepam equivalent medication and buprenorphine/naloxone patients had been given 20 mg. It is known that benzodiazepines such as diazepam or oxazepam, which were commonly administered to the patients in this study may have a slight negative effect on simple RT even among long-term benzodiazepine users [47,48]. Thus, benzodiazepine comedication may have affected the results in RT tasks. It also possible that benzodiazepine comedication would interact differently with methadone than with buprenorphine/naloxone. This possibility is supported by the results of a recent study by Lintzeris et al. showing that mixing 10 or 20 mg of diazepam with a mean 55 mg of methadone had significant detrimental effect on simple RT and focused attention in methadone patients [49]. Mixing the same amounts of diazepam with mean 11 mg of buprenorphine had, however, minimal effect on buprenorphine patients. In sum, we suggest that methadone-treated patients with current benzodiazepine medication tend to show inferior performance in attention tests relative to buprenorphine/naloxone-treated patients with the same characteristics during early OST. Consequently, it cannot be concluded that methadone, as an OST *monotherapy*, would have different effect on attention performance than buprenorphine/naloxone monotherapy.

The good performance of buprenorphine/naloxone patients in simple RT without warning signal along with their reduced performance variance in this measure is a surprising finding because buprenorphine has adverse effect on RTs in healthy controls [9]. In some earlier studies methadone patients with stable doses also have outperformed healthy controls in simple RT [11,50]. In one study, a relatively low dose of methadone of, 33 mg or 16 mg, given during early inpatient opioid withdrawal treatment actually *speeded *RTs of opioid-dependent patients in comparison to placebo condition [12]. In our study methadone patients on 40 mg outperformed methadone patients on 67 mg dose. Together these observations raise the possibility that a low dose of full mu opioid agonist methadone or normal dose of partial mu opioid agonist buprenorphine may have a minimal effect on simple RTs of opioid-dependent patients in OST with high tolerance for these opioids – and also for benzodiazepine comedication.

### Limitations

Cognitive differences between the patient groups may partly relate to differences in their OST drug-tolerance. The majority of the patients in both groups had abused buprenorphine during the recent month. In spite of cross-tolerance to opioids, it is possible that switching from buprenorphine to methadone results in transient cognitive deficits in methadone patients. Thus, the possibility of opioid switching effect in methadone-treated patients during early OST cannot be ruled out. In order to investigate this issue we are currently working on a follow-up study with same patients.

Several psychoactive medications were used nearly similarly in both groups to treat psychiatric comorbidity during the OST initiation. These drugs, such as short acting non-benzodiazepine zopiclone, neuroleptics, anticonvulsant valproate, or antihistamine hydroxyzine may have slight adverse cognitive effects [51-54]. The interactions of OST medications and all these medications warrant for further studies.

All our participants were free from current substance abuse as confirmed by drug screens. Instead, during the recent month preceding the OST patients had used several psychoactive substances. There were no major differences between the uses of these substances within the patient groups. Thus, these substances such as cannabis may explain the differences between the studied OST patient groups only if they have long-term effects and they interact differently with the OST drugs. Therefore, long-term benzodiazepine use may explain part of the *similarities *between the OST patient groups. Long-term benzodiazepine monotherapy has adverse affect on several cognitive functions, which may last at least for six months [55].

Opioid-dependent patients may already differ from the general population in premorbid cognitive functions. Yet, when we controlled for premorbid verbal intelligence by using Verbal IQ estimate as a covariate in ANCOVA procedure, this did not affect the results. Actually, this procedure may be too conservative. A recent study has shown that current smoking (if more than 8 cigarettes per day) affects Verbal IQ [56]. Nearly all of our patients were daily smokers. Thus, verbal IQ differences in these patients may not to be primarily premorbid.

## Conclusion

Both methadone and buprenorphine/naloxone-treated OST patients show deficits in working memory and verbal list learning during the early phase of their treatment. Deficits in attention may be seen only in methadone-treated patients and their impairments may be dose-dependent. Verbal memory deficit may be more extensive in methadone- than in buprenorphine/naloxone-treated patients. Thus, this study further shows that in clinical samples, in which recent benzodiazepine use and benzodiazepine comedication as well as other psychoactive medications are common, methadone-treated patients have more cognitive deficits than buprenorphine- or buprenorphine/naloxone-treated patients. Buprenorphine/naloxone may preserve cognitive function in early OST better than methadone, at least when benzodiazepine comedication is used. Longitudinal studies are warranted to investigate whether this advantage is permanent or is restricted to early OST

## Competing interests

The author(s) declare that they have no competing interests.

## Authors' contributions

PR planned and performed cognitive testing and statistical analysis. He wrote the first version of the manuscript and prepared the final manuscript. HA and MS conceived the idea of this study and advised in manuscript preparation. HK and KW participated in the design of the study and in manuscript preparation. CF carried out psychiatric investigations. All authors read and accepted the final manuscript.

## Pre-publication history

The pre-publication history for this paper can be accessed here:


